# Capacity building in health care professions within the Gulf cooperation council countries: paving the way forward

**DOI:** 10.1186/s12909-019-1513-2

**Published:** 2019-03-14

**Authors:** Javaid I. Sheikh, Sohaila Cheema, Karima Chaabna, Albert B. Lowenfels, Ravinder Mamtani

**Affiliations:** 1 Office of the Dean, Weill Cornell Medicine-Qatar, Doha, Qatar; 2 Institute for Population Health, Weill Cornell Medicine-Qatar, Doha, Qatar; 3 Department of Surgery and Family Medicine, New York Medical College, Valhalla, NY USA

**Keywords:** Capacity building, Continuing education, Continuing professional development, Human resource development, Health care workforce development, GCC countries

## Abstract

**Background:**

There is a worldwide shortage of health care workers. This problem is particularly severe in the Gulf Cooperation Council (GCC) countries because of shortages in certain medical disciplines, due to a lack of nationally-trained professionals and a less developed educational system compared to other high income countries. Consequently, GCC countries are heavily dependent on an expatriate health care workforce; a problem exacerbated by high turnover. We discuss challenges and potential strategies for improving and strengthening capacity building efforts in health care professions in the GCC.

**Main text:**

In the GCC, there are 139 schools providing professional health education in medicine, dentistry, pharmacy, nursing, midwifery, and other specialties. Health education school density reported for the GCC countries ranges between 2.2 and 2.8 schools per one million inhabitants, except in Oman where it is 4.0 per one million inhabitants. The GCC countries rely heavily on expatriate health professionals. The number of physicians and nurses in the GCC countries are 2.1 and 4.5 per 1000 respectively, compared to 2.8 and 7.9 among member countries of the Organisation for Economic Cooperation and Development (OECD). Interestingly, the number of dentists and pharmacists is higher in the GCC countries compared to OECD countries. A nationally trained health care workforce is essential for the GCC countries. Physiotherapy and occupational therapy are two identified areas where growth and development are recommended. Custom-tailored continuing medical education and continuing professional development (CPD) programs can augment the skills of health practitioners, and allow for the expansion of their scope of practice when warranted.

**Conclusion:**

Capacity building can play an essential role in addressing the major health challenges and improving the overall quality of health care in the region. Efforts aimed at increasing the number of locally-trained graduates and developing and implementing need-based CPD programs are vital for capacity building and lifelong learning in health care professions.

**Electronic supplementary material:**

The online version of this article (10.1186/s12909-019-1513-2) contains supplementary material, which is available to authorized users.

## Introduction

In 2016, the World Health Organization (WHO) developed the Global Strategy on Human Resources for Health: Workforce 2030 [1], due to the worldwide shortage of health care workers, which they considered as a potential crisis [2]. The WHO estimated that the worldwide health care workforce had a shortfall of seven million professionals in 2013, and predicted it would reach 18 million by 2030 [1]. Furthermore, the WHO views the unequal distribution of the health care workforce to be a major public health problem. The shortage of health care workers and their unequal geographical distribution are prominently evident in the Gulf Cooperation Council (GCC) countries, namely Bahrain, Kuwait, Oman, Qatar, Saudi Arabia, and the United Arab Emirates (UAE). The extremely rapid economic growth of the Gulf countries during the twentieth century, following the discovery of new energy sources, created opportunities but also presented challenges in managing health care systems [3, 4].

One feature of the shortage of workforce in the GCC countries is a small number of nationally-trained professionals [5]. Consequently, GCC countries are heavily dependent on an expatriate health care workforce, a problem exacerbated by high turnover [1, 5]. In 2014, the densities of physicians, dentistry personnel, and pharmaceutical personnel were 2.2, 0.9, and 1.2 per 1000 population, respectively, compared to 2.8, 0.4, and 0.8 across the Organisation for Economic Cooperation and Development (OECD) member countries (Table 1) [4, 6]. The number of nurses and midwives in the GCC countries is particularly concerning, with a density of only 4.6 per 1000, compared to 8.0 in the OECD countries. Physiotherapy and occupational therapy education are lacking in the GCC countries and require special attention.

**Table 1:** Density of health care workforce (per 1000) in 2014

Location	Physicians	Nurses and midwives	Dentistry personnel	Pharmaceutical personnel
Gulf Cooperation Council^a^	2.193	4.588	0.886	1.189
Bahrain	0.939	2.445	0.249	0.161
Kuwait	1.949	4.729	0.596	0.483
Oman	1.541	3.345	0.185	0.348
Qatar	1.964	5.7	0.572	0.931
Saudi Arabia	2.568	5.207	0.398	0.701
United Arab Emirates	1.558	3.061	3.1	3.68
Member countries of the Organisation for Economic Cooperation and Development^b^	2.800	7.982	0.4	0.807
Canada (2015)	2.539	9.842	1.253	0.98
Australia (2015)	3.496	12.37	0.578	0.847
Norway (2015)	4.385	17.824	0.851	0.741
Japan (2014)	2.367	11.241	0.797	1.704
Mexico (2013)	2.071	2.509	0.117	–
Sweden (2013)	4.107	11.892	0.805	0.755
Turkey (2014)	1.749	3.200	0.297	0.351
United Kingdom (2015)	2.806	8.436	0.535	0.84
United States (2013)	2.554	9.884	–	0.887

Data source: Global Health Observatory data repository [4]

^a^Estimated population-weighted average, using population size estimated by the United Nations [6]

^b^Estimated population-weighted average for all the member countries of the OECD, using population size estimated by the United Nations [6]

Due to the heavy reliance on expatriate health care professionals in the GCC, cultural mismatch between the patient and health care provider is an additional challenge [7]. It is estimated that populations belonging to more than 100 cultures are living in close proximity in the GCC [8]. Efforts in the GCC are underway to address cultural competence; one such example is the cultural competence education program at the Institute for Population Health’s Center for Cultural Competence in Health Care at Weill Cornell Medicine-Qatar (WCM-Q) [8].

The health care delivery landscape has changed dramatically in the GCC during the last few decades. Patient and practitioner priorities have also changed. The epidemic of chronic diseases in the GCC countries will require innovative approaches to management and treatment. Along with health care reform, there is therefore a need for a broad curricular reform to upgrade the existing health education programs within the GCC countries. Challenges associated with reforming education programs include a) the rapidly expanding base of medical knowledge, b) the conflict between ‘top down’ lecture systems and more flexible and innovative ‘flipped’ classroom teaching, and c) the growing disconnect between the traditional curricula with reductive biological basis and the increasing prevalence of chronic diseases, which have strong social determinants. Even when such challenges are identified, the cost, time, and effort required to upgrade the educational systems can present significant hurdles.

The workforce shortage and associated challenges within the GCC affords an opportunity to transform health care systems by appropriately targeted investments and relevant education advancement of existing health care workers [9]. As an example, for physicians, high quality training programs are of limited value if postgraduate training programs for students and continuing professional development (CPD) programs for professionals are not aligned, invested in, or simply do not exist. In the GCC countries, all health care professionals are required to pursue CPD or continuing medical education (CME) for re-licensure. The challenge is to develop appropriate programs that address the needs of the GCC countries with their unique demographics. For GCC countries to achieve the United Nations Sustainable Development Goals by 2030 [10], it is essential to evaluate health care capacity in two critical areas: 1) adequacy of the health care workforce, and 2) profession and specialty specific undergraduate, postgraduate, and CME/CPD education programs.

Consequently, supporting capacity building activities and programs aimed at cultivating and augmenting the ability of health professionals and students to perform competently are essential and much-needed in the GCC. Broadly, capacity building efforts include three essential components: 1) acquiring requisite skills, 2) improving these skills over time, and 3) retaining updated skills [11–13]. These efforts should also focus on organizational development, resource allocation, building partnerships within and outside GCC countries, and developing leadership pathways [13, 14]. In this report we discuss the challenges and potential strategies for improving and strengthening capacity building efforts in health care professions in the GCC. Our discussion is presented in three parts: 1) planning and assessment, 2) infrastructure for health profession schools, and 3) health care workforce capacity building. In the section on health care work capacity building we discuss, debate, and make general recommendations concerning a) the adequacy of the health care workforce, b) CME and CPD, c) the role of regulatory authorities in training health care professionals, d) prominent examples of education-based capacity building efforts in the region, and e) present additional evidence suggesting that health care capacity in the GCC can be strengthened by developing links to non-GCC organizations.

## Discussion

### Planning and assessment

In 2016, the WHO Regional Office for the Eastern Mediterranean organized a meeting on the strategic framework for health workforce development in the region to discuss workforce challenges [15] and emphasized the need to transform health care education and effectively regulate issues surrounding adequacy of health care workforce in the region [16]. The WHO recognized four dimensions that define health care workforce performance: availability, competence, responsiveness, and productivity [2]. The findings of the meeting elaborated further that health care workforce competence, which encompasses the combination of technical knowledge, skills, and behaviors, could be positively influenced by supportive supervision coupled with audit, feedback, and lifelong learning [2]. Although, an adequately and appropriately trained health care workforce is essential, there is currently a lack of sufficient training capacity [17].

In 2017, the WHO reported that strengthening the health system within the GCC countries should include capacity building for the health care workforce [18]. This process includes mapping and assessing the current health care workforce, developing a national strategy, transforming the health care system according to the need, and establishing a recruitment and management system. GCC countries should plan their health care system by focusing on building human capacity at all levels, including academia [19].

Capacity building can play an essential role in addressing the major health challenges and improving the quality of health care in the region. Several steps have been taken to plan and or assess capacity building programs and activities in the GCC countries.

Following the success of the Nursing Strategy 2013–2015, the Nursing and Midwifery Strategy 2015–2018 was launched by Hamad Medical Corporation (HMC) in Qatar, under the leadership of the Ministry of Public Health [20]. The strategy aims to facilitate effective, high quality, evidence-based, and compassionate patient-centered care, recognizing the essential role of nurses and midwives in the region. Through the Nursing Strategy 2013–2015, the Qatari Nursing and Midwifery Development and Mentoring Office was launched to support health care recruitment, continuing education, and participation of HMC’s nurses and midwives in research.

In Saudi Arabia, the Ministry of Health is responsible for strategic planning. As part of the country’s wider strategic National Transformation Program 2020, efforts are being made to radically change the structure and function of the Saudi health care system [21]. This program also focuses on improving infrastructure, however less focus has been placed on health care professional education [19]. Gaps between training, knowledge, and skills of physicians and nurses, and the identified needs to address adolescent health issues, have been reported for Saudi Arabia [22]. These gaps must be addressed. Furthermore, the readiness of Saudi Arabia for health care organizational change has been questioned [19].

Monitoring and evaluation of the GCC health care system transformation should be a key component to measure its effectiveness. Furthermore, implementing contractual agreements between health care providers and the public regulators to align strategy, performance, and accountability should be an additional strategy to assess performance management. In the UAE, mandatory standardized indicators for improving health care quality were developed by the Department of Health in Abu Dhabi (HAAD) [23]. Similarly, in Qatar, the Health Services Performance Agreements initiative was launched by the Ministry of Public Health with all public, private, and semi-governmental hospitals and primary health care centers [24]. The positive impact of standardized health care quality indicators for directing action on quality improvement has been demonstrated by the OECD [25].

The GCC countries aim is to strengthen the health care workforce by developing the training of nationals in all health care professional categories to become self-sufficient [18, 26]. For some health care professions – such as nursing – training sufficient numbers of nationals could be a major challenge [18].

### Infrastructure for health profession schools

In the GCC there are 139 schools that provide professional health care education, including medical, dental, pharmaceutical, nursing, public health, and other allied health education programs at undergraduate and postgraduate levels; the number of schools in each GCC country varies and is proportional to the country’s population (Table 2 and Additional file 1). Differences in the geographical distribution of health care schools merit attention. In Bahrain and Qatar, there is a need to establish a college of dentistry, so that these two countries will not rely exclusively on international dental graduates. There are several nursing schools in the GCC countries, however, schools for other health professionals are lacking or inadequate.

**Table 2:** Density of health care school’s workforce (per 1000) in 2017

Location	Number (%)	Density per one million inhabitants	Missing programs
Gulf Cooperation Council	139 (100)	2.6	–
Bahrain	3 (2.1)	2.2	Dental college and occupational therapy and physiotherapy programs
Kuwait	11 (7.9)	2.8	Midwifery program
Oman	18 (12.9)	4.0	Occupational therapy program
Qatar	6 (4.3)	2.7	Dental college and occupational therapy program
Saudi Arabia	77 (55.4)	2.5	–
United Arab Emirates	24 (17.3)	2.6	Occupational therapy and midwifery programs

To develop adequate health care workforces, GCC countries should focus on establishing new health care schools specializing in under-resourced disciplines, and scaling up the training capacity of their existing schools. In time, this will reduce the shortfall in nationally-trained health care professionals such as nurses, and the subsequent dependency on an expatriate workforce [18].

Local accreditation of educational institutions is essential, and international accreditation is proving to be an invaluable tool in encouraging the development of higher quality health care programs [27, 28]. In the GCC, to ensure quality medical education, the Medical Deans’ Sub-Committee of Accreditation assess GCC countries’ medical schools, guided by standards developed by the World Federation for Medical Education [29].

Building research infrastructure regionwide to develop and implement evidence-based health care is vital. Population health research for example, can identify major health challenges, which helps policymakers in prioritizing health programs while planning for public health and health care. Clinical research contributes to improving health care. The GCC countries are contributing to medical research, which is evident by publications in peer-reviewed medical journals [30]. In the GCC countries, undergraduate medical students can partake in funded research, contribute to research activities, and attend local scientific conferences [31]. In Saudi Arabia, undergraduate medical students demonstrate positive research trends resulting from a research-oriented environment in the medical schools [32]. In Qatar, to build a knowledge-based society supported by the Qatar National Research Strategy 2012’s vision, medical and nursing students are actively encouraged to participate in research [33].

### Health care workforce capacity building

#### Adequacy of health care workforce

The developing high-income countries of the GCC must develop robust capacity-building programs for their health care workforce if they are to realize their ambition of a high-quality health care system. On average, the GCC countries have a higher density of pharmaceutical and dental personnel than across the OECD countries, including countries with some of the best health care systems in the world (Table 1). The average density of physicians and nurses, on the other hand, is lower in the GCC than across the OECD countries.

The high density of dentistry personnel in the GCC is driven by a particularly high rate in the UAE (3.1 per 1000), which is significantly higher than all of the OECD countries listed in Table 1. In Oman, the dental workforce has been growing since 2012, when the Oman Dental College was inaugurated [34]. However, the need for additional dental workforce has been identified in Oman to reach levels found in the OECD. Increasing and retaining Oman Dental College graduates are key to addressing this deficit.

A substantial shortage of nurses and midwives has been demonstrated in the six GCC countries. Their density is far from reaching the levels of the OECD countries (about half of the average density in the OECD countries). We observe that the countries with some of the best health care systems are heavily dependent on the contribution of nurses and midwives.

The profession of physiotherapy has been identified (2013) as an area to be prioritized for development in Kuwait [35] and in the other GCC countries. Our Google search identified 21, 20, 20, and 15 physiotherapy centers available in Qatar, Bahrain, Kuwait, and Oman, respectively for adult and pediatric patients. It is interesting to note that in the UAE, the physiotherapy centers are only located in Abu-Dhabi (eight centers) and Dubai (12 centers), while none exist in the other five Emirate States. Similarly, in Saudi Arabia, most of these centers (53) are located mainly in the major cities of Riyadh, Jeddah, and Dammam.

The Health Professionals Workforce Plan 2012–2022 for Australia, advocates that ‘more of the same is no longer the answer’ [36], suggesting that there is an optimal density of health care professionals for a country, and anything above this will not necessarily strengthen its health care system. Policymakers should plan for the ‘right people’, with the ‘right skills’, in the ‘right place’ [36].

#### CME/CPD programs

Fundamental elements for building capacity in health care professionals through CME/CPD programs are summarized in Table 3. The preliminary requirement for any CME/CPD program is to identify existing needs and gaps and build on programs consistent with national health strategies.

**Table 3:** Fundamental elements recommended for capacity building through CME/CPD programs in the countries of the Gulf Cooperation Council

Main goals	Special topics to be emphasized	Potential barriers
• Identify existing challenges and existing gaps based on overall long-term goals of country • Strengthen existing skills and knowledge of the current workforce • Strengthening human and institutional resources • Strengthen leadership by encouraging personal and professional development • Support innovation and technology • Develop new – or adhere to existing – health guidelines	• Critical thinking • Preventive medicine • Personalized medicine (genetics) • Health informatics • Control of lifestyle diseases • Self-care: avoiding burnout	• Financial limitations • Unrealistic business plan • Reluctance to seek or accept advice from consultants • Failure to obtain or act on patient feedback • Poorly motivated staff • Unsatisfactory interpersonal relationships between staff members. • Rapid staff turnover

In 2014, the WHO Regional Office for the Eastern Mediterranean Region recommended CME/CPD activities as an important method to engage in lifelong learning [37]. Hence, we encourage the development of CME/CPD programs to build health care capacity in the GCC countries. The process in strengthening CME/CPD programs should include: 1) country-specific needs assessment, 2) post-remediation programs, 3) assessment of the CME/CPD activity impact at the health care professional and patient levels, 4) assessing the number of trained health care professionals still working in the country at mid- and long-term, and 5) identification of areas requiring additional improvement. This continuous process is essential for the health care workforce to maintain existing skills and to keep up with the rapid advances in areas such as lifestyle diseases, genetics, and personalized medicine. Additionally, we recommend that each GCC country provides appropriate, culturally sensitive CME/CPD activities to ensure effective patient-centered care that takes into account the cultural diversity that characterizes these countries [7].

Capacity building must focus not only on the challenges of prevalent lifestyle-driven chronic diseases, such as diabetes and obesity, but also on newly emerging infectious diseases [38–41]. A multi-disciplinary approach that includes capacity building is the ideal method to prepare the health care workforce to tackle these issues [39]. Capacity building education programs, which include task shifting from physicians to other health professionals, can lower cost and expand capacity in resource-short areas [42]. Task shifting in the GCC countries will strengthen interdisciplinary and interprofessional collaboration, leading to improved health outcomes for individuals, communities, and the wider population.

#### Regulatory authorities for CME/CPD programs and capacity building of health care professionals

In addition to undergraduate and postgraduate programs, CME/CPD programs are essential to build capacity in health care and ensure the system is patient-oriented, more efficient, and clearly addresses identified needs and gaps. There are eight national health authorities in the GCC countries (Table 4) [43–48]. The aim of these authorities is to promote continuous quality improvement by: 1) setting standards, 2) providing an external evaluation of compliance of institutions/programs against those standards, and 3) proposing a remediation or an improvement process following the review of an application [27].

**Table 4:** National health authorities evaluating health institutions and health care CME/CPD programs in the GCC countries

Country	Institution for accreditation	Year established
Bahrain	• Training Directorate – Ministry of Health – Kingdom of Bahrain (https://www.moh.gov.bh/Services/ContinuingMedicalEducation?lang=ar) [44]	NS
Kuwait	• Center for Continuing Education and Professional Development of the Kuwait Institute of Medical Specialization (https://www.kims-cepd.org/website/about.php) [45]	NS
Oman	• Oman Medical Specialty Board (http://www.omsb.org/) [46]	2006
Qatar	• The Registration and Licensing Department in Qatar Council for Healthcare Practitioners (QCHP, http://www.qchp.org.qa/en/Pages/Registration.aspx) [47]	2013
United Arab Emirates	• Ministry of Health (MOH, http://www.mohap.gov.ae/en/Pages/default.aspx) • Health Authority - Abu Dhabi (HAAD, https://www.haad.ae/haad/) • Dubai Health Authority (DHA, https://www.dha.gov.ae/en/pages/dhahome.aspx) [43]	NS NS 2008
Saudi Arabia	• Saudi Commission for Health Specialties (https://www.scfhs.org.sa/en/Pages/default.aspx) [48]	1992

*NS* year not specified

#### Country-specific examples of training and capacity building efforts in the GCC

In this section we provide several examples where GCC countries have developed local programs, designed to strengthen country-specific capacity building.

Bahrain and Saudi Arabia have a collaborative initiative to develop CME programs focusing on infectious diseases. The 2nd Gulf Congress of Clinical Microbiology and Infectious Diseases [49] was held in November 2017 in Bahrain and accredited by the Saudi Commission for Health Specialties. Topics included antimicrobial resistance, transplant infectious disease, infection prevention and control, among others. Health care professionals from both countries were able to benefit from this initiative by presenting their own research, being exposed to the latest research in the fields, and claiming credits for re-licensure. Supporting health care professionals in referring to and conducting research is an effective strategy towards evidence-based health care.

In addition to collaboration between GCC countries to build health care capacities, Gulf countries also collaborate with the WHO for guidance aimed at strengthening health care systems and improving population health. For instance, Oman has worked closely with the WHO to develop a primary health care approach to addressing public health issues such as infant mortality [34]. In Kuwait, the WHO Global Oral Health Programme is working with the WHO Collaborating Centre for Primary Oral Health Care, Kuwait University, to reinforce the development of appropriate models for primary oral health care – a major health need in the country [50]. Similar collaborative initiatives with the WHO might be a valid strategy to addressing public health issues in GCC countries where there is an identified need.

While local programs to develop health care capacity may not address all the needs in the GCC, capacity building strategies can also send health care professionals overseas to gain experience, knowledge, and skills. For instance, by 2015 the Oman Ministry of Health had granted 104 scholarships to teaching staff from the Health Sciences Institutes to study abroad, half of which were for Ph.D. programs [51]. Enabling health care professionals to study overseas is a relatively quick fix to develop and sustain a highly-qualified health care workforce while awaiting development of local educational capacities.

In Qatar, to advance its tripartite mission of education, research, and clinical excellence, WCM-Q established the Institute for Population Health (IPH). One of the purposes of IPH is to strengthen health professionals’ capacity through high quality educational and training programs. In 2017, the IPH held the Building Capacity in Healthcare Professions Symposium. Following a preliminary needs assessment, the symposium hosted six workshops, followed by six plenary sessions. The main symposium conclusions were that people (social capital) and communication skills are the critical components of capacity building, and that capacity building programs should be inter-professional, inter-disciplinary, and emphasize teamwork, inclusivity, collaboration, and systems thinking.

Several programmatic activities have been developed in Saudi Arabia to enhance the knowledge and skills of health care professionals [52]. For instance, King Abdulaziz Medical City, Central Region (KAMC-CR) invested in training staff in patient care quality and safety. Sustaining these efforts is currently the primary challenge that KAMC-CR faces [52].

The GCC has a young, diverse, and multicultural population of over 50 million people, with varying levels of education [47]. A substantial proportion of the GCC countries’ population aged over 15 years are non-nationals, reaching over 80% in Qatar and the UAE [8]. Qatar’s capital, Doha, is recognized as hosting speakers of 190 languages [9]. Non-nationals constitute a majority of both the population and health care professionals in these countries [7, 48]. Health professions educational programs such as nursing, should continue to focus on issues surrounding provision of culturally sensitive healthcare. A study including nursing students from Saudi Arabia and Oman demonstrated how undertrained students are in cultural competence [49]. Capacity building efforts in cultural competence are underway in the region to improve interaction between patients and health care providers and amongst health care staff who do not originate from the same culture [9]. In Qatar, to address the cultural and linguistic challenges due to the diverse patient population, WCM-Q developed the Center for Cultural Competence in Health Care, which provides education in medical interpretation and cultural competence [9]. Financial support and public health decisions are required to scale-up medical interpreters’ recruitment in all health care centers and to make cultural competence programs mandatory for all health care professionals who serve diverse populations.

#### Evidence for strengthening healthcare capacity by developing links to organizations outside the GCC

In this section, we demonstrate that developing links between organizations located in the GCC countries and those located in non-GCC countries has been a great opportunity to strengthen health care capacity. There are several types of international educational partnerships between various institutions in the GCC and non-GCC countries that already exist and involve capacity building (Table 4). Partnerships include international scholarships, premedical programs for undergraduate students, partial institutional link at a program level, consulting agreement between two institutions, establishment of new completely affiliated medical school in a recipient country, and physician exchange programs at institutional level; each has its strengths and weaknesses [53–58].

An example of one global partnership is the health care training facility WCM-Q, founded in 2001 and affiliated with the well-established and respected Weill Cornell Medicine in New York. This is an innovative and integrative approach to building health care capacity in Qatar, with the overall mission of training physicians who can later assume local leadership positions in health care, public health, and research, and, most importantly, teach and mentor the next generation of medical students and residents. The first class graduated in 2008 after completing the six-year curriculum. Between 2008 and 2018, 325 medical students have graduated. The benefit of WCM-Q in strengthening the medical capacity in Qatar is already underway; several graduates have returned to the country after their residency in the United State or Canada, to work at either the Hamad Medical Corporation, the major health care provider within Qatar, or at WCM-Q, to participate in advancing health education and training to develop the health care capacity in the country. The number of Qatari students in the program has increased to 35%.

Similar innovative and integrative approaches have been established at the University of Calgary in Qatar, affiliated to the University of Calgary’s Faculty of Nursing in Canada, which offers a nursing program (57 nursing graduates in 2016) [59], and at the Royal College of Surgeons in Ireland – Medical University of Bahrain, a constituent university of the Royal College of Surgeons in Ireland, which offers training programs in medicine, nursing, and midwifery [60]. Bringing high quality training to build health care capacity locally is a long-term strategy that we believe will increase the quality of health care in the GCC countries.

International scholarship programs are a flexible and integrative approach to building health care capacity at undergraduate and postgraduate levels. In some instances, the cost may be less than the cost of developing comparable local training facilities. The Dubai Harvard Foundation for Medical Research in UAE funds up to 50% of tuition in a selected list of postgraduate programs located in the USA or in UAE, such as Global Clinical Scholars Research Training and Introduction to Clinical Research Training-Dubai. This Foundation gives priority to UAE nationals but candidates from the GCC, the Middle East and North Africa region, and non-national UAE residents are also considered. Building capacity through international scholarship has its limitations, due to the uncertainty related to the return of students to the GCC countries. A better strategy may be the scholarship programs designed to support nationally trained health care professionals in local health care training facilities affiliated to well-established institutions from outside the GCC. The AlMabarrah AlKhalifia Foundation’s Rayaat Scholarship program in Bahrain has a partnership with Qatar [61]. Her Highness Shaikha Moza bint Hamad Medical Grant was launched in 2016 to fund the tuition fees for one applicant to the undergraduate medicine program at the Royal College of Surgeons in Ireland – Bahrain. Only Bahraini high school graduates who meet the specified criteria are eligible to apply, which supports capacity building of national health care professionals.

Collaboration between GCC and non-GCC organizations can be also be illustrated at a CPD/CME level. Physician exchange programs are a flexible, integrative, and innovative approach for rapid, cost-effective technology and knowledge transfer. Specialists from the Children’s National Medical Center in Washington, D.C. frequently travel to Kuwait, Qatar, UAE, and Saudi Arabia for educational exchange sessions at regional hospitals, health care agencies, and conferences [55]. The center also provides a children’s worldwide telemedicine program providing clinical consultations in Kuwait, Qatar, and UAE, among other countries. Medical professionals from the GCC are received at the Sheikh Zayed Campus for Advanced Children’s Medicine in Washington, D.C. to share their expertise with the center’s medical and research staff. With respect to continuing training for nurses, the Massachusetts General Hospital in Boston provides a program for nurses around the world to improve their skills at the hospital [62]. These are examples of existing partnership programs with organizations outside of the GCC that have served to enhance health care capacity within the region. These holistic approaches have been successful in responding to local needs because they are flexible, integrated, and innovative. As is evident from Table 5 these partnerships of cooperation are not without challenges.

**Table 5:** Examples of cooperative international medical relationships developed in the GCC countries

Program type	Description	Advantages	Disadvantages
International scholarships. *Example: Dubai Harvard Foundation for Medical Research* [53]	Selected students from the GCC sent to host country to obtain medial training.	Minimal expenditure on support structures and faculty. Flexible and integrated.	Students may endeavor to stay in host country. Limited overall improvement to existing health profile.
Premedical programs for undergraduate students. *Example: New York University Abu Dhabi* [54]	Students in existing or new university can take premedical course.	Avoids expensive costs of maintaining a full medical school. Integrated and innovative.	Students may face problems selecting and entering an approved medical school.
Partial institutional link at a program level. *Johns Hopkins Medicine International and Al Rahda Hospital* [56, 63] *University of Pittsburgh Medical Center and Hamad Medical Corporation* [57]	Donor school agrees to establish and supervise a particular program in recipient institution.	Works well when recipient institution has well-organized facilities but lacks a specific program. Flexible, integrative, and innovative.	Country-specific cultural differences can impair program effectiveness.
Consulting agreement between two institutions. *Example: collaborative project between medical schools of Aberdeen University and the UAE University* [58]	Donor institution signs agreement covering agreed-upon deliverables to upgrade recipient institution.	Recipient institution can benefit from organizational skills and experience of donor organization without committing to long-term investment in personnel with requisite skills. Flexible, integrative, and innovative.	Resentment from personnel in recipient institution may interfere with agreed-upon objectives.
Establishment of new, completely affiliated health educational school in recipient country *Example: Weill Cornell Medicine - Qatar* [64] *and The Royal College of Surgeons in Ireland – Bahrain* [60]	Donor institution agrees to develop a completely integrated satellite school with same standards and courses as the existing school in the donor country.	Offers high quality education to selected students. Provides unique opportunity to strengthen the foundation of the recipient country’s health system. Integrated and innovative.	Expensive. Both institutions must make a long-term commitment (not flexible).
Physician exchange programs at institutional level. *Example: GCC countries and Children’s National Hospital* [55]	Physicians with special skills travel and work in distant schools or hospitals.	Method for rapid, cost-effective knowledge and skill transfer. Especially effective if there is a two-way physician exchange (flexible, integrative, and innovative).	Logistical problems such as language and cultural differences. Immediate benefit, but duration of improvement unsure.

## Conclusion

Recent attempts to include health care professionals from multiple disciplines (physicians, nurses, pharmacists, dentists, midwives, others) in the GCC’s health care workforce and increasing the number of locally-trained graduates are important steps to address challenges with the type and supply of health care professionals. Along those lines, establishing models of team-based training and diversifying health care curricula and CME/CPD programs are vital for capacity building endeavors. These steps will decrease the reliance on an expatriate health care workforce and improve the quality of health care in the region. Similarly, building capacity at undergraduate and postgraduate training levels are also steps in the right direction. CME/CPD programs are a vital component for capacity building in health care professions as they transform the existing health care systems by training the workforce to address identified population health needs.

To increase the professional competence (skills and knowledge), culturally-sensitive capacity building efforts should be based on country-specific needs assessments that include an educational approach focused on problem-based and competence-based learning. Capacity building can play a vital role in addressing the major health challenges of this region, which include: an aging population, the increase of chronic non-communicable diseases, the continuing threat of infectious disease, the rising concern of mental health issues, and inequity in women’s health. Capacity-building efforts within and outside of academic institutions can play a significant role in addressing gaps in the education of health professionals and strengthening of health care systems in the GCC countries.

## Additional file

Additional file 1: List of identified health care schools in the countries of the Gulf Cooperation Council. List of identified health care schools in the six countries of the Gulf Cooperation Council, namely Bahrain, Kuwait, Oman, Qatar, United Arab Emirates, and Saudi Arabia. (PDF 42 kb)

## Abbreviations

CME/CPD: continuing medical education/continuing professional development
GCC: Gulf Cooperation Council
IPH: Institute for Population Health
KAMC-CR: King Abdulaziz Medical City, Central Region
UAE: United Arab Emirates
USA: United State of America
WCM-Q: Weill Cornell Medicine-Qatar
WHO: World Health Organization

## References

[1] Draft global strategy on human resources for health: workforce 2030 [http://apps.who.int/gb/ebwha/pdf_files/WHA69/A69_38-en.pdf]. Accessed 12 Mar 2019.

[2] World Health Organization (2006). World health report 2006: working together for health.

[3] World Health Organization (2015). World health Statistics 2015.

[4] Global Health Observatory data repository [http://apps.who.int/gho/data/view.main.92100]. Accessed 12 Mar 2019.

[5] Exploring the Problem of Scarcity of Nurses in Underserved Areas in the Middle East: Factors, Reasons and Incentives for Recruitment and Retention [http://www.who.int/alliance-hpsr/projects/americanuniversity_hrhincentives/en/]. Accessed 12 Mar 2019.

[6] Total Population - Both Sexes. De facto population in a country, area or region as of 1 July of the year indicated. Figures are presented in thousands. World Population Prospects: The 2015 Revision [https://esa.un.org/unpd/wpp/Download/Standard/Population/]. Accessed 12 Mar 2019.

[7] Chaabna K, Cheema S, Mamtani R (2017). Migrants, healthy worker effect, and mortality trends in the Gulf cooperation council countries. PLoS One.

[8] Elnashar M, Abdelrahim H, Fetters MD (2012). Cultural competence springs up in the desert: the story of the center for cultural competence in health care at Weill Cornell Medical College in Qatar. Acad Med.

[9] Darzi A, Evans T (2016). The global shortage of health workers-an opportunity to transform care. Lancet.

[10] Sustainable development goals. 17 goals to transform the world [http://www.un.org/sustainabledevelopment/sustainable-development-goals/]. Accessed 12 Mar 2019.

[11] Crisp B, Swerissen H, Duckett S (2000). Four approaches to capacity building in health: consequences for measurement and accountability. Health Promot Int.

[12] Mumghamba EG, Joury E, Fatusi O, Ober-Oluoch J, Onigbanjo RJ, Honkala S (2015). Capacity building and financing Oral health in the African and Middle East region. Adv Dent Res.

[13] Hawe P, King L, Noort M, Jordens C, Lloyd B (1999). Indicators to help with capacity building in health promotion.

[14] Fikri M (2017). Roadmap 2017-2021: stronger organization and better response to the needs of member states in the eastern Mediterranean region. East Mediterr Health J.

[15] World Health Organization. A strategic framework for health workforce development in the Eastern Mediterranean Region. East Mediterr Health J. 2017;23(5):388–89. 30378676

[16] World Health Organization (2016). Summary report on the Meeting on the strategic framework for health workforce development in the Eastern Mediterranean Region.

[17] Heller RF, Chongsuvivatwong V, Hailegeorgios S, Dada J, Torun P, Madhok R, Sandars J. Capacity-building for public health: http://peoples-uni.org. Bull World Health Organ 2007, 85(12):930–934. 10.2471/BLT.07.044388 PMC2636296 18278252

[18] Country Cooperation Strategy at a glance. Kuwait [http://apps.who.int/iris/bitstream/handle/10665/136906/ccsbrief_kwt_en.pdf;jsessionid=B9311C600CA404814614AD9504F5C5CC?sequence=1]. Accessed 12 Mar 2019.

[19] Alharbi MF (2018). An analysis of the Saudi health-care system's readiness to change in the context of the Saudi National Health-care Plan in vision 2030. Int J Health Sci (Qassim).

[20] Hamad Medical Corporation Nursing and Midwifery Strategy 2015-2018 [https://www.hamad.qa/EN/Hospitals-and-services/Nursing/Nursing-Strategy/nursing_strategy_15_18/Documents/Nursing-Strategy-2015-18-E.pdf]. Accessed 12 Mar 2019.

[21] National Transformation Program 2020 [http://www.vision2030.gov.sa/sites/default/files/NTP_En.pdf]. Accessed 12 Mar 2019.

[22] AlBuhairan FS, Olsson TM (2014). Advancing adolescent health and health services in Saudi Arabia: exploring health-care providers' training, interest, and perceptions of the health-care needs of young people. Adv Med Educ Pract.

[23] ‘JAWDA’ to Raise Quality of Healthcare Services in the Emirate of Abu Dhabi [https://www.haad.ae/haad/tabid/58/Mid/417/ItemID/451/ctl/Details/Default.aspx]. Accessed 12 Mar 2019.

[24] Al-Katheeri H, El-Jardali F, Ataya N, Abdulla Salem N, Abbas Badr N, Jamal D (2018). Contractual health services performance agreements for responsive health systems: from conception to implementation in the case of Qatar. Int J Qual Health Care.

[25] Rotar AM, van den Berg MJ, Kringos DS, Klazinga NS (2016). Reporting and use of the OECD health care quality indicators at national and regional level in 15 countries. Int J Qual Health Care.

[26] The 8th five-year plan for health development (2011-2015). The National Strategic Plan [https://www.mah.se/upload/five_year_plan_for_health_development_2011-2015%20Oman.pdf]. Accessed 12 Mar 2019.

[27] What is Accreditation? [https://www.achs.org.au/about-us/what-we-do/what-is-accreditation/]. Accessed 12 Mar 2019.

[28] van Zanten M, Norcini JJ, Boulet JR, Simon F (2008). Overview of accreditation of undergraduate medical education programmes worldwide. Med Educ.

[29] Gulf Co-operation Council Medical Colleges Deans’ Committee. Recommendations and Guidelines on Minimum Standards for Establishing and Accrediting Medical Schools in the Arabian Gulf Countries. Bahrain: Secretariat of the GCC Medical Colleges Deans’ Committee; 2001.

[30] Meo SA, Hassan A, Aqil M, Usmani AM (2015). Medical education research in GCC countries. BMC Med Educ.

[31] Sayedalamin Z, Halawa TF, Baig M, Almutairi O, Allam H, Jameel T, Gazzaz ZJ, Atta H (2018). Undergraduate medical research in the Gulf cooperation council (GCC) countries: a descriptive study of the students' perspective. BMC Res Notes.

[32] Obeidat AS, Alhaqwi AI, Abdulghani HM (2015). Reprioritizing current research trends in medical education: a reflection on research activities in Saudi Arabia. Med Teach.

[33] Research @ UCQ [http://www.ucalgary.edu.qa/fs/research]. Accessed 12 Mar 2019.

[34] Gallagher JE, Manickam S, Wilson NH (2015). Sultanate of Oman: building a dental workforce. Hum Resour Health.

[35] MacPherson MM, MacArthur L, Jadan P, Glassman L, Bouzubar FF, Hamdan E, Landry MD (2013). A SWOT analysis of the physiotherapy profession in Kuwait. Physiother Res Int.

[36] Health Professionals Workforce Plan 2012-2022 [https://www.health.nsw.gov.au/workforce/hpwp/Publications/health-professionals-workforce-plan-2012.pdf]. Accessed 12 Mar 2019.

[37] Report on the Intercountry meeting on strengthening medical education in the Eastern Mediterranean Region [https://apps.who.int/iris/bitstream/handle/10665/253424/IC_Meet_Rep_2015_EN_16357.pdf?sequence=1&isAllowed=y]. Accessed 12 Mar 2019.

[38] Aly M, Elrobh M, Alzayer M, Aljuhani S, Balkhy H (2017). Occurrence of the Middle East respiratory syndrome coronavirus (MERS-CoV) across the Gulf corporation council countries: four years update. PLoS One.

[39] Ahmed J, Bouloy M, Ergonul O, Fooks A, Paweska J, Chevalier V, Drosten C, Moormann R, Tordo N, Vatansever Z, et al. International network for capacity building for the control of emerging viral vector-borne zoonotic diseases: ARBO-ZOONET. Euro Surveill. 2009;14(12). 19341603

[40] Zumla A, Goodfellow I, Kasolo F, Ntoumi F, Buchy P, Bates M, Azhar EI, Cotten M, Petersen E (2016). Zika virus outbreak and the case for building effective and sustainable rapid diagnostics laboratory capacity globally. Int J Infect Dis.

[41] Cheema S, Maisonneuve P, Weber I, Fernandez-Luque L, Abraham A, Alrouh H, Sheikh J, Lowenfels AB, Mamtani R (2017). Knowledge and perceptions about Zika virus in a Middle East country. BMC Infect Dis.

[42] Martinez-Gonzalez NA, Tandjung R, Djalali S, Rosemann T (2015). The impact of physician-nurse task shifting in primary care on the course of disease: a systematic review. Hum Resour Health.

[43] Healthcare professionals qualification requirements 2014 https://s3-ap-southeast-1.amazonaws.com/dataflow-dfgcorp/wp-content/uploads/2017/01/06064347/HAAD_PQR_July2011.pdf (PQR)2014–1.pdf]. Accessed 12 Mar 2019.

[44] Continuing Medical Eduction [https://www.moh.gov.bh/Services/ContinuingMedicalEducation?lang=ar]. Accessed 12 Mar 2019.

[45] About Us [https://www.kims-cepd.org/website/about.php]. Accessed 12 Mar 2019.

[46] Oman Medical Specialty Board [http://omsb.org/File_Publications/files_20150312094907015_b2ce7816-9bf4-48f4-ab05-7050e67ae5d3.pdf]. Accessed 12 Mar 2019.

[47] Registration & Licensing Department. World Class Standards. Qualified Practitioners. [http://www.qchp.org.qa/en/Pages/Registration.aspx]. Accessed 12 Mar 2019.

[48] Continuing Medical Education and Program Accreditation [http://www.scfhs.org.sa/en/CME-ADRP/Pages/default.aspx]. Accessed 12 Mar 2019.

[49] Second Gulf Congress of Clinical Microbiology and Infectious Disease [http://gccmid.org/]. Accessed 12 Mar 2019.

[50] Petersen PE (2014). Strengthening of oral health systems: oral health through primary health care. Med Princ Pract.

[51] Health Education in the Sultanate of Oman [https://educouncil.gov.om/en/page.php?scrollto=start&id=87]. Accessed 12 Mar 2019.

[52] Arabi YM, Taher S, Berenholtz SM, Alamry A, Hijazi R, Alatassi A, Marini AM (2013). Building capacity for quality and safety in critical care: A roundtable discussion from the second international patient safety conference in April 9-11, 2013, Riyadh, Saudi Arabia. Ann Thorac Med.

[53] Dubai Harvard Foundation for Medical Research [http://www.dhfmr.hms.harvard.edu/]. Accessed 12 Mar 2019.

[54] NYU Abu Dhabi [http://nyuad.nyu.edu/en/]. Accessed 12 Mar 2019.

[55] Knowledge sharing [http://www.childrensnational.ae/knowledge-sharing].

[56] Johns Hopkins Medicine International and Al Rahbda Hospital [https://www.hopkinsmedicine.org/international/international_affiliations/middle_east/al_rahba_hospital.html]. Accessed 12 Mar 2019.

[57] UPMC helps to create new trauma service in Qatar [http://www.upmc.com/media/NewsReleases/2007/Pages/upmc-helps-to-create-new-trauma-service-in-qatar.aspx]. Accessed 12 Mar 2019.

[58] Brebner EM, Brebner JA, Norman JN, Brown PA, Ruddick-Bracken H, Lanphear JH (1997). Intercontinental postmortem studies using interactive television. J Telemed Telecare.

[59] University of Calgery in Qatar [http://www.ucalgary.edu.qa/]. Accessed 12 Mar 2019.

[60] Programmes [http://www.rcsi-mub.com/Programmes]. Accessed 12 Mar 2019.

[61] Annual report 2016–2017 [https://www.rcsi.com/bahrain/about-rcsi-bahrain/annual-report]. Accessed 12 Mar 2019.

[62] Global Nursing Education Program [http://www.cvent.com/events/global-nursing-education-program/event-summary-fbcab9b08f15403286960f35b964baf6.aspx]. Accessed 12 Mar 2019.

[63] Johns Hopkins Medicine [https://www.hopkinsmedicine.org/international/index.html]. Accessed 12 Mar 2019.

[64] Weill Cornll Medicine-Qatar [https://qatar-weill.cornell.edu/about-us/overview/weill-cornell-medicine-qatar]. Accessed 12 Mar 2019.

