# Supplementary material for: Capacity building in health care professions within the Gulf cooperation council countries: paving the way forward
Source: BMC Med Educ. 2019 Mar 14;19:83. doi: 10.1186/s12909-019-1513-2 (PMC6417223; doi:10.1186/s12909-019-1513-2)
Supplement: Supplementary file 1 — List of identified health care schools in the countries of the Gulf Cooperation Council. List of identified health care schools in the six countries of the Gulf Cooperation Council, namely Bahrain, Kuwait, Oman, Qatar, United Arab Emirates, and Saudi Arabia. (PDF 42 kb) [file 12909_2019_1513_MOESM1_ESM.pdf]

Additional file 1. List of identified healthcare school in the countries of Gulf Cooperation Council

Country	School	Location
**Bahrain**	University of Bahrain, College of Health Sciences (Nursing, Dental Hygiene, Pharmacy, Public Health, Medical Laboratory Technology, Radiography)	Salmaniya
	Arabian Gulf University, College of Medicine and Medical Sciences	Manama
	Royal College of Surgeons in Ireland - Bahrain	Busaiteen
**Kuwait**	Kuwait University, Health Science Center, Faculty of Medicine	Kuwait City
	Kuwait University, Health Science Center, Faculty of Allied Health Sciences	Kuwait City
	Kuwait University, Health Science Center, Faculty of Pharmacy	Kuwait City
	Kuwait University, Health Science Center, Faculty of Dentistry	Kuwait City
	Kuwait University, Health Science Center, Faculty of Public Health	Kuwait City
	Kuwait Institute for Medical Specialization	Kuwait City
	The American University for Medical Sciences-Kuwait, School of Medicine	Kuwait City
	The American University for Medical Sciences-Kuwait, School of Dental Medicine	Kuwait City
	The American University for Medical Sciences-Kuwait, School of Pharmacy	Kuwait City
	The American University for Medical Sciences-Kuwait, College of Health Sciences	Kuwait City
	The College of Nursing	Kuwait City
**Oman**	Al-Dhahira Nursing Institute (regional)	Al-Dhahira
	Oman Medical College	Bawshar
	Ibra Nursing Institute (regional)	Ibra
	Institute of Health Sciences	Matrah
	Oman Dental College	Muscat
	Sultan Qaboos University, College of Medicine and Health Sciences	Muscat
	Sultan Qaboos University, College of Nursing	Muscat
	Oman Nursing Institute	Muscat

	Muscat Nursing Institute	Muscat
	Institutes of Health Sciences, Oman Institute for Health Information Management	Muscat
	Higher College of Technology, College of Pharmacy	Muscat
	Al-Dakhilia Nursing Institute (regional)	Nizwa
	Rustaq Nursing Institute (regional)	Rustaq
	Salalah Nursing Institute (regional)	Salalah
	North Batinah Nursing Institute (regional)	Sohar
	Sohar Nursing Institute (regional)	Sohar
	Sur Nursing Institute (regional)	Sur
	Oman Assistant Pharmacist Institute	Wataya
**Qatar**	Qatar University, College of Medicine	Doha
	Qatar University, College of Pharmacy	Doha
	Qatar University, College of Health Sciences	Doha
	Weill Cornell Medicine-Qatar	Doha
	College of the North Atlantic Qatar, School of Health Sciences	Doha
	University of Calgary in Qatar	Doha
**United Arab Emirates**	New York University Abu Dhabi	Abu Dhabi
	Fatima College of Health Sciences	Abu Dhabi
	Gulf Medical University, College of Medicine	Ajman
	Gulf Medical University, College of Dentistry	Ajman
	Gulf Medical University, College of Pharmacy	Ajman
	Gulf Medical University, College of Health Sciences	Ajman
	Gulf Medical University, College of Graduate studies	Ajman
	United Arab Emirates University, College of Medicine and Health Sciences	Al Ain
	Dubai Medical College for Girls	Dubai
	Mohammed bin Rashid University of Medicine and Health Sciences, College of Medicine	Dubai
	Hamdan Bin Morhammed College of Dental Medicine	Dubai

	The Royal College of Surgeons in Ireland – Dubai	Dubai
	Harvard Medical School for Global Health Delivery – Dubai	Dubai
	Dubai Pharmacy College	Dubai
	International Health And Safety Training Center	Dubai
	Ras al-Khaimah Medical and Health Sciences University, College of Medical Sciences	Ras Al-Khaimah
	Ras al-Khaimah Medical and Health Sciences University, College of Dental Sciences	Ras Al-Khaimah
	Ras al-Khaimah Medical and Health Sciences University, College of Pharmaceutical Sciences	Ras Al-Khaimah
	Ras al-Khaimah Medical and Health Sciences University, College of Nursing	Ras Al-Khaimah
	University of Sharjah, College of Health Sciences	Sharjah
	University of Sharjah, College of Medicine	Sharjah
	University of Sharjah, College of Dental Medicine	Sharjah
	University of Sharjah, College of Pharmacy	Sharjah
	University of Sharjah, Clinical and surgical Training Center	Sharjah
**Saudi Arabia**	King Khalid University College of Medicine and Medical Sciences	Abha
	King Faisal University	Al Ahsa
	Al Baha University, Medical College	Al Baha
	Al Baha University, Faculty of Dentistry	Al Baha
	Al Baha University, Faculty of Clinical Pharmacy	Al Baha
	Al Baha University, Faculty of Applied Medical Sciences	Al Baha
	Sulaiman Alrajhi College of Medicine, Al Bukairiyah	Al Bukayriyah
	Taibah University College of Medicine	Al Madinah
	Majmaah University College of Medicine	Al Majmaah
	King Saud bin Abdulaziz University for Health Sciences, College of Applied Medical Sciences	Al-Ahsa
	Prince Sattam Bin Abdulaziz University College of Medicine, Al-Kharj	Al-Kharj
	Northern Borders University College of Medicine	Arar

	College of Medicine, University of Bisha	Bisha
	Qassim University College of Medicine	Buraidah
	College of Dentistry	Buraidah
	University of Dammam College of Medicine	Dammam
	College of Dentistry (Females)	Dammam
	Mohammed Al-mana College Of Health Science (MACHS),	Dammam
	King Faysal University, College of Clinical Pharmacy	Dammam
	University of Hail College of Medicine	Hail
	Jazan University Faculty of Medicine	Jazan
	College of Dentistry	Jazan
	Jazan University Faculty of Dentistry	Jazan
	Jazan University Faculty of Pharmacy	Jazan
	Jazan University Faculty of Applied Medical Sciences	Jazan
	Jazan University Faculty of Health Sciences	Jazan
	Batterjee Medical College, Medical program	Jeddah
	Ibn Sina National College for Medical Studies, Jeddah	Jeddah
	King Abdulaziz University Faculty of Medicine	Jeddah
	King Saud bin Abdulaziz University for Health Sciences, College of Medicine	Jeddah
	Ibn Sina National Medical College, Dentistry program	Jeddah
	Ibn Sina National Medical College, Pharmacy program	Jeddah
	King Abdulaziz University Faculty of Dentistry	Jeddah
	King Abdulaziz University Faculty of Pharmacy	Jeddah
	Ibn Sina National Medical College, Nursing program	Jeddah
	King Saud bin Abdulaziz University for Health Sciences, College of Applied Medical Sciences	Jeddah
	King Abdulaziz University Faculty of Nursing	Jeddah
	King Abdulaziz University	Jizan
	Al-Majmaah University, College of Medicine	Majmaah
	Umm Al-Qura University College of Medicine and Medical Sciences	Makkah

	Najran University College of Medicine	Najran
	Alfaisal University, College of Medicine	Riyadh
	Al-Farabi Colleges	Riyadh
	Almaarefa College of Medicine, College of Medicine	Riyadh
	Global Colleges College of Medicine	Riyadh
	King Fahad Medical City Faculty of Medicine	Riyadh
	King Saud bin Abdulaziz University for Health Sciences, College of Medicine	Riyadh
	King Saud University College of Medicine	Riyadh
	Princess Nourah Bint Abdulrahman University College of Medicine	Riyadh
	Riyadh Colleges of Dentistry and Pharmacy	Riyadh
	College of Dentistry - BUC	Riyadh
	College of Dentistry	Riyadh
	Batterjee Medical College, Dentistry program	Riyadh
	King Saud bin Abdulaziz University for Health Sciences, College of Dentistry	Riyadh
	King Saud bin Abdulaziz University for Health Sciences, College of Pharmacy	Riyadh
	Batterjee Medical College, Pharmacy program	Riyadh
	King Saud University, College of Pharmacy	Riyadh
	Almaarefa College of Medicine, College of Pharmacy	Riyadh
	Alfaisal University, College of Pharmacy	Riyadh
	Batterjee Medical College, Nursing program	Riyadh
	Batterjee Medical College, Physical therapy program	Riyadh
	Batterjee Medical College, Radiologic Sciences program	Riyadh
	Almaarefa College of Medicine, College of Applied Sciences	Riyadh
	Batterjee Medical College, Healthcare admin program	Riyadh
	King Saud University, College of Nursing	Riyadh
	King Saud bin Abdulaziz University for Health Sciences, College of Public Health and Health Informatics	Riyadh

	King Saud bin Abdulaziz University for Health Sciences, College of Applied Medical Sciences	Riyadh
	King Saud bin Abdulaziz University for Health Sciences, Colleges of Science and Health Professions	Riyadh, Al-Ahsa
	King Saud bin Abdulaziz University for Health Sciences, Colleges of Nursing	Riyadh, Jeddah, Al-Ahsa
	Al-Jouf University, College of Medicine	Sakaka
	Al-Jouf University, College of Dentistry	Sakaka
	Al-Jouf University, College of Pharmacy	Sakaka
	Al-Jouf University, College of Applied Medical Sciences	Sakaka
	Tabuk University	Tabuk
	University of Tabuk Faculty of Medicine	Tabuk
	Taif University College of Medicine	Taif
	Unaizah College of Medicine	Unaizah
